# Supplementary material for: Functional Improvement and Satisfaction with a Wearable Hip Exoskeleton in Community-Living Adults
Source: Healthcare (Basel). 2023 Feb 22;11(5):643. doi: 10.3390/healthcare11050643 (PMC10000834; doi:10.3390/healthcare11050643)
Supplement: Supplementary file 1 [file healthcare-11-00643-s001.zip › healthcare-2053466-supplementary.pdf]

Supplementary Table S1. Regression analysis between general characteristics, usability, and satisfaction

Independent variables	Dependent variables	B	t-value	R^2	p-value
Age	Safety1	-0.008	-2.269	0.340	0.024*
	Safety2	0.000	-0.093	0.036	0.926
	Safety3	-0.009	-1.556	0.022	0.121
	Safety4	-0.003	-1.372	0.020	0.171
	Satisfaction1	-0.027	-4.970	0.146	0.000***
	Satisfaction2	-0.014	-1.656	0.028	0.099
	Satisfaction3	0.006	0.884	0.031	0.378
	Satisfaction4	0.019	2.597	0.082	0.010*
	Satisfaction5	-0.017	-2.568	0.055	0.011*
	Satisfaction6	-0.008	-1.095	0.044	0.275
	Satisfaction7	-0.008	-1.205	0.530	0.230
	Satisfaction8	-0.012	-2.789	0.093	0.006**
	Satisfaction9	-0.009	-1.896	0.037	0.059
	Satisfaction10	0.015	2.730	0.068	0.007**
	Satisfaction11	-0.014	-2.147	0.029	0.033*
	Satisfaction12	-0.027	-3.416	0.074	0.001**
Gender	Safety1	-0.157	-1.389	0.340	0.166
	Safety2	0.061	1.001	0.036	0.318
	Safety3	-0.259	-1.457	0.022	0.146
	Safety4	0.034	0.504	0.020	0.615
	Satisfaction1	-0.013	-0.075	0.146	0.941
	Satisfaction2	-0.131	-0.478	0.028	0.633
	Satisfaction3	-0.022	-0.101	0.031	0.920
	Satisfaction4	0.219	0.961	0.082	0.338
	Satisfaction5	-0.139	-0.650	0.055	0.517
	Satisfaction6	0.122	0.551	0.044	0.582
	Satisfaction7	0.015	0.074	0.530	0.941
	Satisfaction8	-0.132	-1.000	0.093	0.318
	Satisfaction9	-0.269	-1.739	0.037	0.083
	Satisfaction10	0.119	0.698	0.068	0.486
	Satisfaction11	-0.090	-0.424	0.029	0.672
	Satisfaction12	-0.004	-0.015	0.074	0.988
Height	Safety1	0.010	0.283	0.340	0.777
	Safety2	0.017	0.917	0.036	0.360
	Safety3	-0.015	-0.289	0.022	0.773
	Safety4	-0.015	-0.742	0.020	0.459
	Satisfaction1	0.010	0.186	0.146	0.852
	Satisfaction2	-0.076	-0.915	0.028	0.361
	Satisfaction3	-0.050	-0.765	0.031	0.445
	Satisfaction4	0.117	1.703	0.082	0.090
	Satisfaction5	0.006	0.092	0.055	0.927
	Satisfaction6	0.034	0.518	0.044	0.605
	Satisfaction7	0.015	0.241	0.530	0.809
	Satisfaction8	0.053	1.322	0.093	0.187
	Satisfaction9	-0.063	-1.346	0.037	0.180

Independent variables	Dependent variables	B	t-value	R^2	p-value
	Satisfaction10	-0.037	-0.719	0.068	0.473
	Satisfaction11	-0.027	-0.416	0.029	0.678
	Satisfaction12	0.047	0.628	0.074	0.531
Weight	Safety1	-0.022	-0.510	0.340	0.610
	Safety2	-0.020	-0.883	0.036	0.378
	Safety3	0.003	0.044	0.022	0.965
	Safety4	0.019	0.753	0.020	0.452
	Satisfaction1	-0.034	-0.523	0.146	0.601
	Satisfaction2	0.094	0.902	0.028	0.368
	Satisfaction3	0.079	0.969	0.031	0.333
	Satisfaction4	-0.140	-1.627	0.082	0.105
	Satisfaction5	-0.041	-0.504	0.055	0.615
	Satisfaction6	-0.064	-0.760	0.044	0.448
	Satisfaction7	-0.023	-0.301	0.530	0.764
	Satisfaction8	-0.081	-1.615	0.093	0.108
	Satisfaction9	0.062	1.052	0.037	0.294
	Satisfaction10	0.050	0.768	0.068	0.443
	Satisfaction11	0.026	0.323	0.029	0.747
	Satisfaction12	-0.066	-0.696	0.074	0.487
BMI	Safety1	0.049	0.436	0.340	0.664
	Safety2	0.065	1.075	0.036	0.283
	Safety3	-0.040	-0.228	0.022	0.820
	Safety4	-0.053	-0.784	0.020	0.434
	Satisfaction1	0.087	0.508	0.146	0.612
	Satisfaction2	-0.231	-0.842	0.028	0.401
	Satisfaction3	-0.199	-0.926	0.031	0.355
	Satisfaction4	0.368	1.624	0.082	0.106
	Satisfaction5	0.104	0.490	0.055	0.625
	Satisfaction6	0.178	0.811	0.044	0.418
	Satisfaction7	0.088	0.434	0.530	0.664
	Satisfaction8	0.213	1.622	0.093	0.106
	Satisfaction9	-0.173	-1.123	0.037	0.263
	Satisfaction10	-0.113	-0.665	0.068	0.507
	Satisfaction11	-0.070	-0.330	0.029	0.741
	Satisfaction12	0.193	0.775	0.074	0.439
Health condition	Safety1	0.009	0.169	0.340	0.866
	Safety2	-0.012	-0.462	0.036	0.644
	Safety3	-0.034	-0.434	0.022	0.665
	Safety4	0.000	-0.007	0.020	0.995
	Satisfaction1	-0.187	-2.449	0.146	0.015*
	Satisfaction2	-0.029	-0.235	0.028	0.814
	Satisfaction3	-0.117	-1.212	0.031	0.227
	Satisfaction4	0.211	2.071	0.082	0.040*
	Satisfaction5	-0.135	-1.417	0.055	0.158
	Satisfaction6	-0.205	-2.083	0.044	0.038*
	Satisfaction7	-0.109	-1.195	0.530	0.233
	Satisfaction8	-0.154	-2.629	0.093	0.009**

Independent variables	Dependent variables	B	t-value	R^2	p-value
	Satisfaction9	-0.064	-0.921	0.037	0.358
	Satisfaction10	0.063	0.833	0.068	0.406
	Satisfaction11	0.041	0.440	0.029	0.660
	Satisfaction12	-0.036	-0.323	0.074	0.747
Activity	Safety1	0.091	1.377	0.340	0.170
	Safety2	0.047	1.318	0.036	0.189
	Safety3	0.242	2.335	0.022	0.020*
	Safety4	-0.028	-0.715	0.020	0.475
	Satisfaction1	0.074	0.740	0.146	0.460
	Satisfaction2	-0.104	-0.651	0.028	0.516
	Satisfaction3	-0.010	-0.080	0.031	0.936
	Satisfaction4	-0.076	-0.576	0.082	0.565
	Satisfaction5	0.113	0.905	0.055	0.366
	Satisfaction6	0.067	0.519	0.044	0.604
	Satisfaction7	0.250	2.090	0.530	0.038*
	Satisfaction8	0.032	0.422	0.093	0.673
	Satisfaction9	-0.003	-0.036	0.037	0.971
	Satisfaction10	0.148	1.481	0.068	0.140
	Satisfaction11	0.161	1.307	0.029	0.193
	Satisfaction12	-0.155	-1.062	0.074	0.290
experience of fall	Safety1	0.053	0.372	0.340	0.710
	Safety2	0.070	0.914	0.036	0.362
	Safety3	-0.107	-0.478	0.022	0.633
	Safety4	0.022	0.259	0.020	0.796
	Satisfaction1	0.542	2.496	0.146	0.013*
	Satisfaction2	0.309	0.889	0.028	0.375
	Satisfaction3	0.300	1.098	0.031	0.274
	Satisfaction4	-0.454	-1.578	0.082	0.116
	Satisfaction5	0.419	1.546	0.055	0.123
	Satisfaction6	0.102	0.367	0.044	0.714
	Satisfaction7	0.452	1.746	0.530	0.082
	Satisfaction8	-0.284	-1.700	0.093	0.091
	Satisfaction9	0.179	0.916	0.037	0.361
	Satisfaction10	0.228	1.055	0.068	0.293
	Satisfaction11	0.132	0.491	0.029	0.624
	Satisfaction12	0.308	0.972	0.074	0.332

Note. *p<0.05, **p<0.01, ***p<0.001.

Supplementary Table S2. Regression analysis between usability, satisfaction, and physical function

Independent variables	Dependent variables	B	t-value	R^2	p-value
Age	SPPB	-0.022	-4.139	0.368	0.000***
	FSST	0.031	2.94	0.284	0.004**
	TUG	0.01	0.968	0.232	0.334
	10MWT	-0.002	-1.76	0.262	0.080
	6MWT	-0.277	-0.637	0.211	0.525
	ΔSPPB	0.003	1.057	0.198	0.292
	ΔFSST	-0.003	-0.574	0.151	0.567
	ΔTUG	-0.012	-1.887	0.114	0.061
	Δ10MWT	0.001	1.307	0.139	0.193
	Δ6MWT	-0.199	-1.101	0.113	0.272
Gender	SPPB	-0.001	-0.007	0.368	0.995
	FSST	-0.73	-2.496	0.284	0.013*
	TUG	-0.01	-0.036	0.232	0.971
	10MWT	-0.043	-1.128	0.262	0.261
	6MWT	-15.559	-1.272	0.211	0.205
	ΔSPPB	0.064	0.826	0.198	0.410
	ΔFSST	-0.414	-2.773	0.151	0.006**
	ΔTUG	0.058	0.323	0.114	0.747
	Δ10MWT	-0.002	-0.067	0.139	0.947
	Δ6MWT	2.193	0.43	0.113	0.668
Height	SPPB	-0.042	-0.948	0.368	0.344
	FSST	0.062	0.703	0.284	0.483
	TUG	-0.018	-0.212	0.232	0.832
	10MWT	0.002	0.217	0.262	0.829
	6MWT	1.073	0.292	0.211	0.771
	ΔSPPB	0.023	0.976	0.198	0.330
	ΔFSST	-0.009	-0.198	0.151	0.844
	ΔTUG	-0.047	-0.873	0.114	0.384
	Δ10MWT	0.006	0.85	0.139	0.396
	Δ6MWT	-1.847	-1.205	0.113	0.230
Weight	SPPB	0.058	1.049	0.368	0.296
	FSST	-0.144	-1.298	0.284	0.196
	TUG	-0.004	-0.033	0.232	0.974
	10MWT	-0.001	-0.036	0.262	0.971
	6MWT	-0.682	-0.147	0.211	0.883
	ΔSPPB	-0.026	-0.874	0.198	0.383
	ΔFSST	-0.025	-0.448	0.151	0.655
	ΔTUG	0.054	0.804	0.114	0.422
	Δ10MWT	-0.006	-0.649	0.139	0.517
	Δ6MWT	2.516	1.302	0.113	0.195
BMI	SPPB	-0.173	-1.186	0.368	0.237
	FSST	0.396	1.358	0.284	0.176
	TUG	0.07	0.246	0.232	0.806
	10MWT	-0.009	-0.239	0.262	0.811
	6MWT	-0.286	-0.023	0.211	0.981

Independent variables	Dependent variables	B	t-value	R^2	p-value
	ΔSPPB	0.069	0.889	0.198	0.375
	ΔFSST	0.065	0.435	0.151	0.664
	ΔTUG	-0.132	-0.743	0.114	0.458
	Δ10MWT	0.011	0.478	0.139	0.633
	Δ6MWT	-6.161	-1.212	0.113	0.227
Health condition	SPPB	-0.077	-1.158	0.368	0.248
	FSST	0.265	1.996	0.284	0.047*
	TUG	0.311	2.393	0.232	0.018**
	10MWT	-0.03	-1.739	0.262	0.084
	6MWT	-5.429	-0.977	0.211	0.330
	ΔSPPB	0.026	0.727	0.198	0.468
	ΔFSST	0.032	0.477	0.151	0.634
	ΔTUG	0.026	0.319	0.114	0.750
	Δ10MWT	-0.011	-1.01	0.139	0.314
	Δ6MWT	3.056	1.319	0.113	0.189
Level of activity	SPPB	0.142	1.653	0.368	0.100
	FSST	0.194	1.13	0.284	0.260
	TUG	-0.174	-1.036	0.232	0.302
	10MWT	0.054	2.448	0.262	0.015*
	6MWT	15.522	2.165	0.211	0.032*
	ΔSPPB	-0.051	-1.11	0.198	0.268
	ΔFSST	0.136	1.553	0.151	0.122
	ΔTUG	0.029	0.279	0.114	0.780
	Δ10MWT	0.007	0.485	0.139	0.628
	Δ6MWT	-0.852	-0.285	0.113	0.776
Experience of fall	SPPB	-0.534	-2.849	0.368	0.005**
	FSST	0.604	1.614	0.284	0.108
	TUG	0.755	2.06	0.232	0.041*
	10MWT	-0.111	-2.282	0.262	0.024*
	6MWT	-31.515	-2.013	0.211	0.045*
	ΔSPPB	0.376	3.784	0.198	0.000***
	ΔFSST	0.184	0.962	0.151	0.337
	ΔTUG	0.446	1.953	0.114	0.052
	Δ10MWT	-0.056	-1.871	0.139	0.063
	Δ6MWT	7.67	1.175	0.113	0.241
Safety1	SPPB	0.184	1.987	0.368	0.048*
	FSST	-0.065	-0.352	0.284	0.725
	TUG	0.206	1.14	0.232	0.256
	10MWT	-0.002	-0.099	0.262	0.922
	6MWT	-10.448	-1.35	0.211	0.179
	ΔSPPB	-0.13	-2.654	0.198	0.009**
	ΔFSST	0.005	0.051	0.151	0.960
	ΔTUG	0.083	0.736	0.114	0.462
	Δ10MWT	0.021	1.421	0.139	0.157
	Δ6MWT	0.035	0.011	0.113	0.991
Safety2	SPPB	-0.158	-0.898	0.368	0.370
	FSST	0.071	0.201	0.284	0.841

Independent variables	Dependent variables	B	t-value	R^2	p-value
	TUG	0.066	0.19	0.232	0.849
	10MWT	-0.01	-0.21	0.262	0.834
	6MWT	3.326	0.226	0.211	0.822
	ΔSPPB	0.104	1.115	0.198	0.266
	ΔFSST	0.429	2.383	0.151	0.018**
	ΔTUG	0.126	0.587	0.114	0.558
	Δ10MWT	-0.04	-1.415	0.139	0.159
	Δ6MWT	7.484	1.219	0.113	0.224
Safety3	SPPB	0.119	2.048	0.368	0.042*
	FSST	-0.083	-0.712	0.284	0.477
	TUG	-0.227	-1.999	0.232	0.047*
	10MWT	0.014	0.927	0.262	0.355
	6MWT	8.217	1.694	0.211	0.092
	ΔSPPB	-0.002	-0.075	0.198	0.940
	ΔFSST	-0.042	-0.715	0.151	0.476
	ΔTUG	-0.015	-0.216	0.114	0.829
	Δ10MWT	-0.016	-1.702	0.139	0.090
	Δ6MWT	1.925	0.952	0.113	0.342
Safety4	SPPB	0.338	2.235	0.368	0.027*
	FSST	-0.013	-0.044	0.284	0.965
	TUG	-0.075	-0.255	0.232	0.799
	10MWT	-0.034	-0.877	0.262	0.382
	6MWT	3.319	0.263	0.211	0.793
	ΔSPPB	0.017	0.208	0.198	0.836
	ΔFSST	0.248	1.608	0.151	0.109
	ΔTUG	0.123	0.67	0.114	0.504
	Δ10MWT	-0.02	-0.834	0.139	0.405
	Δ6MWT	3.668	0.697	0.113	0.487
Satisfaction1	SPPB	0.237	3.406	0.368	0.001**
	FSST	-0.163	-1.171	0.284	0.243
	TUG	-0.286	-2.097	0.232	0.037*
	10MWT	0.015	0.853	0.262	0.395
	6MWT	9.798	1.682	0.211	0.094
	ΔSPPB	-0.072	-1.946	0.198	0.053
	ΔFSST	-0.165	-2.327	0.151	0.021*
	ΔTUG	-0.116	-1.367	0.114	0.173
	Δ10MWT	0.003	0.257	0.139	0.797
	Δ6MWT	-2.248	-0.926	0.113	0.356
Satisfaction2	SPPB	-0.009	-0.217	0.368	0.829
	FSST	-0.046	-0.542	0.284	0.588
	TUG	-0.045	-0.533	0.232	0.595
	10MWT	0.012	1.072	0.262	0.285
	6MWT	-1.231	-0.345	0.211	0.731
	ΔSPPB	-0.021	-0.932	0.198	0.352
	ΔFSST	0.028	0.644	0.151	0.521
	ΔTUG	0.01	0.198	0.114	0.843
	Δ10MWT	0.003	0.478	0.139	0.633

Independent variables	Dependent variables	B	t-value	R^2	p-value
	Δ6MWT	-0.932	-0.626	0.113	0.532
Satisfaction3	SPPB	-0.041	-0.775	0.368	0.439
	FSST	0.109	1.036	0.284	0.301
	TUG	-0.035	-0.337	0.232	0.737
	10MWT	0.008	0.59	0.262	0.556
	6MWT	5.007	1.136	0.211	0.257
	ΔSPPB	0.042	1.488	0.198	0.138
	ΔFSST	0.033	0.613	0.151	0.541
	ΔTUG	-0.013	-0.196	0.114	0.845
	Δ10MWT	-0.009	-1.028	0.139	0.305
	Δ6MWT	-0.152	-0.083	0.113	0.934
Satisfaction4	SPPB	-0.023	-0.491	0.368	0.624
	FSST	0.21	2.272	0.284	0.024**
	TUG	0.183	2.023	0.232	0.044*
	10MWT	-0.02	-1.709	0.262	0.089
	6MWT	-6.849	-1.768	0.211	0.079
	ΔSPPB	-0.001	-0.058	0.198	0.954
	ΔFSST	0.008	0.168	0.151	0.867
	ΔTUG	0.029	0.521	0.114	0.603
	Δ10MWT	0.004	0.476	0.139	0.635
	Δ6MWT	1.636	1.014	0.113	0.312
Satisfaction5	SPPB	-0.053	-0.945	0.368	0.346
	FSST	-0.023	-0.203	0.284	0.839
	TUG	-0.078	-0.706	0.232	0.481
	10MWT	0.01	0.706	0.262	0.481
	6MWT	1.974	0.42	0.211	0.675
	ΔSPPB	-0.011	-0.38	0.198	0.704
	ΔFSST	-0.037	-0.654	0.151	0.514
	ΔTUG	0.025	0.369	0.114	0.713
	Δ10MWT	0.009	1.061	0.139	0.290
	Δ6MWT	-0.724	-0.37	0.113	0.712
Satisfaction6	SPPB	-0.037	-0.674	0.368	0.501
	FSST	-0.101	-0.913	0.284	0.362
	TUG	0.11	1.015	0.232	0.312
	10MWT	0.001	0.1	0.262	0.921
	6MWT	-0.273	-0.059	0.211	0.953
	ΔSPPB	0.05	1.699	0.198	0.091
	ΔFSST	-0.016	-0.288	0.151	0.773
	ΔTUG	0.057	0.842	0.114	0.401
	Δ10MWT	-0.008	-0.966	0.139	0.335
	Δ6MWT	3.56	1.852	0.113	0.066
Satisfaction7	SPPB	-0.073	-1.227	0.368	0.221
	FSST	0.146	1.223	0.284	0.223
	TUG	0.192	1.644	0.232	0.102
	10MWT	-0.001	-0.08	0.262	0.937
	6MWT	-5.315	-1.064	0.211	0.289
	ΔSPPB	0.028	0.895	0.198	0.372

Independent variables	Dependent variables	B	t-value	R^2	p-value
	ΔFSST	0.01	0.163	0.151	0.871
	ΔTUG	0.042	0.58	0.114	0.563
	Δ10MWT	0.019	2.001	0.139	0.047*
	Δ6MWT	-3.19	-1.532	0.113	0.127
Satisfaction8	SPPB	-0.025	-0.301	0.368	0.764
	FSST	-0.073	-0.446	0.284	0.656
	TUG	0.079	0.491	0.232	0.624
	10MWT	-0.03	-1.403	0.262	0.162
	6MWT	-3.567	-0.522	0.211	0.602
	ΔSPPB	0.046	1.05	0.198	0.295
	ΔFSST	0.059	0.712	0.151	0.477
	ΔTUG	0.083	0.836	0.114	0.404
	Δ10MWT	-0.011	-0.833	0.139	0.406
	Δ6MWT	4.839	1.699	0.113	0.091
Satisfaction9	SPPB	0.062	0.842	0.368	0.401
	FSST	-0.115	-0.789	0.284	0.431
	TUG	0.039	0.274	0.232	0.785
	10MWT	-0.001	-0.071	0.262	0.944
	6MWT	1.753	0.287	0.211	0.775
	ΔSPPB	0.056	1.432	0.198	0.154
	ΔFSST	0.073	0.976	0.151	0.330
	ΔTUG	0.121	1.353	0.114	0.178
	Δ10MWT	-0.018	-1.568	0.139	0.119
	Δ6MWT	1.962	0.77	0.113	0.442
Satisfaction10	SPPB	0.006	0.1	0.368	0.920
	FSST	-0.009	-0.072	0.284	0.943
	TUG	-0.048	-0.384	0.232	0.701
	10MWT	0.004	0.258	0.262	0.796
	6MWT	-2.422	-0.455	0.211	0.650
	ΔSPPB	0.002	0.047	0.198	0.962
	ΔFSST	0.021	0.325	0.151	0.746
	ΔTUG	-0.127	-1.638	0.114	0.103
	Δ10MWT	0.005	0.48	0.139	0.632
	Δ6MWT	-0.363	-0.164	0.113	0.870
Satisfaction11	SPPB	0.019	0.349	0.368	0.727
	FSST	-0.111	-1.025	0.284	0.307
	TUG	0.071	0.673	0.232	0.502
	10MWT	-0.019	-1.39	0.262	0.166
	6MWT	-1.19	-0.263	0.211	0.793
	ΔSPPB	-0.019	-0.665	0.198	0.507
	ΔFSST	-0.071	-1.292	0.151	0.198
	ΔTUG	0.044	0.662	0.114	0.509
	Δ10MWT	-0.007	-0.851	0.139	0.396
	Δ6MWT	0.237	0.126	0.113	0.900
Satisfaction12	SPPB	-0.016	-0.323	0.368	0.747
	FSST	0.167	1.686	0.284	0.093
	TUG	-0.037	-0.381	0.232	0.703

Independent variables	Dependent variables	B	t-value	R^2	p-value
	10MWT	0.011	0.833	0.262	0.406
	6MWT	-0.727	-0.176	0.211	0.860
	ΔSPPB	-0.009	-0.341	0.198	0.734
	ΔFSST	0.028	0.546	0.151	0.586
	ΔTUG	-0.004	-0.059	0.114	0.953
	Δ10MWT	0.002	0.215	0.139	0.830
	Δ6MWT	1.923	1.116	0.113	0.266

Note. *$p<0.05$, **$p<0.01$, ***$p<0.001$, SPPB=Short physical performance battery; FSST=Four square step test; TUG=Timed up and go; 10MWT=10-meter walking test; 6MWT=6-minute walking test.
